# Microenvironment-Dependent Phenotypic Changes in a SCID Mouse Model for Malignant Mesothelioma

**DOI:** 10.3389/fonc.2013.00203

**Published:** 2013-08-09

**Authors:** Eva Darai-Ramqvist, Gustav Nilsonne, Carmen Flores-Staino, Anders Hjerpe, Katalin Dobra

**Affiliations:** ^1^Department of Laboratory Medicine, Division of Pathology, Karolinska Institutet, Stockholm, Sweden; ^2^Department of Microbiology, Tumor and Cell Biology, Karolinska Institutet, Stockholm, Sweden; ^3^Department of Clinical Neuroscience, Karolinska Institutet, Stockholm, Sweden; ^4^Stress Research Institute, Stockholm University, Stockholm, Sweden

**Keywords:** SCID mice, epithelioid, sarcomatoid, chromosome 3, mesothelioma, differentiation

## Abstract

**Background and Aims:** Malignant mesothelioma is an aggressive, therapy-resistant tumor. Mesothelioma cells may assume an epithelioid or a sarcomatoid phenotype, and presence of sarcomatoid cells predicts poor prognosis. In this study, we investigated differentiation of mesothelioma cells in a xenograft model, where mesothelioma cells of both phenotypes were induced to form tumors in severe combined immunodeficiency mice.

**Methods:** Xenografts were established and thoroughly characterized using a comprehensive immunohistochemical panel, array comparative genomic hybridization (aCGH) of chromosome 3, fluorescent *in situ* hybridization, and electron microscopy.

**Results:** Epithelioid and sarcomatoid cells gave rise to xenografts of similar epithelioid morphology. While sarcomatoid-derived xenografts had higher growth rates, the morphology and expression of differentiation-related markers was similar between xenografts derived from both phenotypes. aCGH showed a convergent genotype for both xenografts, resembling the original aggressive sarcomatoid cell sub-line.

**Conclusion:** Human mesothelioma xenografts from sarcomatoid and epithelioid phenotypes converged to a similar differentiation state, and genetic analyses suggested that clonal selection in the mouse microenvironment was a major contributing factor. This thoroughly characterized animal model can be used for further studies of molecular events underlying tumor cell differentiation.

## Background

Malignant mesothelioma is a tumor that arises from mesothelial cells lining the serosal cavities. Mesothelial cells display an intermediate epithelial-mesenchymal phenotype, and characteristically co-express both epithelial and mesenchymal markers, such as cytokeratins and vimentin (1). Mesothelial progenitor cells are able to switch between different cell phenotypes depending on the local environment, and they can differentiate to epithelioid or mesenchymal phenotypes during tissue homeostasis *in vivo* (2–6). This plasticity of mesothelial cells and the potential to differentiate between these two phenotypes is retained also in malignant mesothelioma cells *in vitro* (7–9). This differentiation into stable epithelioid or fibroblast-like/sarcomatoid phenotypes can be induced *in vitro* by serum growth factors (10). Thus, mesothelioma cells provide a useful model for identifying critical mechanisms involved in the regulation of tumor cell differentiation.

Histological phenotype is the most important prognostic marker for malignant mesothelioma; predominance of a sarcomatoid component indicates worse prognosis and therapy resistance (11–13). Mesothelioma cells of the two different phenotypes have distinct gene expression signatures (14–17). We have previously shown that sarcomatoid mesothelioma cells overexpress growth factor receptors and associated binding proteins, whereas epithelioid mesothelioma cells overexpress tumor promoting factors involved in differentiation, metabolism, and proteasome activation (14). Overall, the expression profile of the epithelioid cell-line reflects a more differentiated tumor. Sarcomatoid mesothelioma cells, however, have a profile associated with growth factors and genes which may contribute to the particularly unfavorable prognosis of sarcomatoid tumors.

Epithelioid and sarcomatoid phenotypes also differ in drug sensitivity profiles (18, 19). A deeper understanding of trans-differentiation between epithelioid and sarcomatoid phenotypes is relevant for the development of therapeutics, and should be taken into consideration when designing *in vitro* studies and establishing animal models. Mouse models of malignant mesothelioma have been previously described; however, only a few studied the differentiation state of the xenografts (20–22).

In this study, we describe the establishment of a mouse xenograft model for malignant mesothelioma, where cells of epithelioid and sarcomatoid phenotypes were injected subcutaneously and concurrently into Severe Combined Immunodeficiency (SCID) mice. We report extensive characterization of the resulting xenografts using immunohistochemistry, electron microscopy, and chromosome 3 array comparative genomic hybridization (aCGH); with particular regard to the differentiation state and genotype of the original cells and their corresponding xenografts. Genetic analyses were performed on chromosome 3, which is one of the most rearranged chromosomes in solid tumors (23). Several earlier studies have highlighted rearrangements on chromosome 3 in malignant mesothelioma (24–27), and we have previously shown that the frequency of rearrangements at specific break-points on chromosome 3 correlates to the degree of genomic instability in cancer cells (28, 29).

## Materials and Methods

### Cells and culture conditions

**Figure 1:**
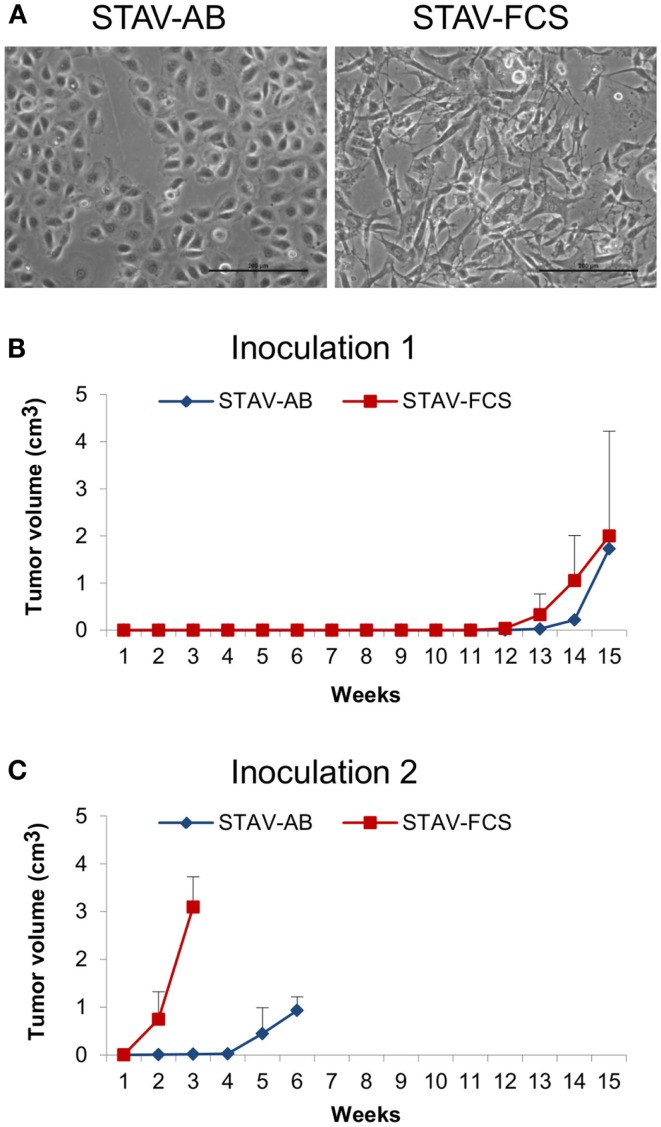
**Mesothelioma cells and xenografts**. **(A)** Phase-contrast micrographs showing epithelioid cobblestone-like morphology in STAV-AB cells, and elongated fibroblast-like morphology in STAV-FCS cells. Scale bars are 200 μm. **(B,C)** Tumor volume after inoculation of mesothelioma cells into SCID mice displaying epithelioid (STAV-AB) or sarcomatoid (STAV-FCS) phenotypes, respectively. After establishment, tumors were serially passed through a second set of mice.

This study was performed using a well-established model system for malignant mesothelioma differentiation, consisting of STAV-AB and STAV-FCS sub-lines (Figure 1A). Cells were originally derived from a single tumor, and subsequently induced to differentiate into stable epithelioid (STAV-AB) and sarcomatoid (STAV-FCS) phenotypes, respectively, by altering the serum composition (10). STAV-AB cells were grown in Gibco RPMI 1640 medium (Invitrogen) and 10% human AB serum, whereas STAV-FCS cells were grown in the same medium and 10% fetal calf serum. All cells were grown in 75 cm^2^ tissue culture flasks (Sarstedt, Newton, MA, USA) at 37°C in 5% CO_2_. Both cell sub-lines have been thoroughly characterized by genome-wide screening with regard to their differentiation state and their molecular signature (14, 30).

### Establishment of xenografts in SCID mice

For establishment of the xenograft model epithelioid (STAV-AB) and sarcomatoid (STAV-FCS) mesothelioma cells with distinct phenotypes (Figure 1A) were inoculated into SCID mice. One million cells in 0.2 ml Iscove’s modified Dulbecco’s medium were inoculated subcutaneously or intraperitoneally into 6-week old mice. Three independent experiments were performed with a total number of 19 mice. In the first two experiments, cells were injected subcutaneously and intraperitoneally and the animals were monitored for 8 weeks with no signs of tumor formation. In the third experiment, mice were inoculated subcutaneously with STAV-AB in one flank and STAV-FCS cells in the other flank. When the xenografts reached the maximal ethically tolerable tumor burden the animals were sacrificed and the tumors were excised and after homogenization serially passaged to the next generation of SCID mice. The xenografts used for subsequent analyses were randomly selected from the second generations of tumors.

### Chromosome 3 array comparative genomic hybridization

A set of 174 commercially available BAC/PAC clones were selected for a chromosome 3 specific array from BACPAC Resources Center, Children’s Hospital Oakland, Oakland, USA^1^; all clones were mapped by fluorescence *in situ* hybridization (FISH), they were at least partially sequenced, their localization was approved using the UCSC database^2^, they were chromosome 3 specific, non-chimeric, and covered the whole chromosome with a resolution of ∼1 Mb. Total genomic DNA was isolated using the GenElute mammalian genomic DNA miniprep kit (Sigma-Aldrich, Germany). DNA labeling, hybridization and post-hybridization processing, scanning, and image analysis were performed as previously described (28). The average and coefficient of variation of fluorescence ratios for each measurement point were calculated. Data points displaying a coefficient of variation >5% between at least two of the replica spots were excluded from further analysis. The average of fluorescence ratios from autosomal controls was used in the normalization of data in each hybridization experiment. All aCGH data have been deposited in NCBI’s Gene Expression Omnibus (31), and are accessible through GEO accession number GSE48019^3^.

### Immunohistochemistry and histochemistry

Based on our previous molecular characterization (14, 17, 30), a panel of antibodies was selected to stain the xenografts. Calretinin (DAKO), was included as a marker for mesothelial lineage. Epithelioid differentiation was investigated using MNF-116 (DAKO), epithelial membrane antigen (EMA) (DAKO), Cam5.2, high molecular weight cytokeratins (CK-HMW) (Novocastra Laboratories), cytokeratin 7 (Novocastra Laboratories), cytokeratin 8 (Novocastra Laboratories), E-cadherin (Novocastra Laboratories), and syndecan-1 (DAKO). Syndecan-2 (Santa Cruz Biotechnology) and vimentin (DAKO) were used as markers for mesenchymal differentiation. We also included the proliferation marker MIB-1 (DAKO), annexin-II, Heat Shock Protein-47 (HSP47), integrin αVβ5 (Chemicon), and thioredoxin reductase 1 (TrxR1) (Upstate), which have been shown to be differentially expressed between the two mesothelioma phenotypes (17, 18, 32). Isotype IgG controls were used to estimate the non-specific binding of target primary antibodies to cell surface antigens. Vascular density in xenografts was demonstrated by silver staining of reticulin fibers according to Gordon and Sweets (33, 34). Vascular density in xenografts was measured by point-counting, comparing different areas using two-sided Student’s *t*-tests, taking α = 0.05.

Immunostainings were performed on cytospin preparations from cell lines, and on formalin-fixed paraffin-embedded sections from xenografts (*n* = 1 per cell sub-line). After deparaffinization and hydration, tissue sections were microwave-treated for antigen retrieval in 10 mM sodium citrate buffer, pH 6.0 for 15 min. Staining was performed following the microwave streptavidin immunoperoxidase (MSIP) protocol on a DakoCytomation TechMate™ instrument (DAKO, Copenhagen, Denmark). Non-specific binding was blocked by 0.5% BSA-TBST (ChemMate™ Detection Kit, DAKO) and endogenous peroxidase activity was abolished by the ChemMate™ Peroxidase-Blocking Solution (S 2023, DAKO). Reaction products were visualized with the streptavidin-biotin-peroxidase method using diaminobenzidine as substrate-chromogen and with hematoxylin as counterstain. Immunohistochemical staining intensities were evaluated semi-quantitatively and referred to as weak (+), moderate (++), or strong (+++), respectively, by two experienced pathologists (Katalin Dobra and Anders Hjerpe). Discrepant cases were re-evaluated and discussed to reach consensus. Vessels were identified in silver stained slides, for morphometrical determination of microvessel density and vessel volume density, respectively.

### Fluorescent in situ hybridization

Chromosomal ploidy patterns were obtained by FISH analysis, using the UroVysion^®^ kit (Abbot). This probe set is used in clinical practice for detecting p16 and the centromeres of chromosomes 3, 7, and 17 (35). Hybridization reactions were performed on cytospin preparations of cultured cells and on formalin-fixed paraffin-embedded sections of xenograft tissue. Four additional chromosome 3 PAC/BAC probes (Rp11-266L17/RP11-89H10 and RP11-356G4/RP11-13k6) were used to analyse the STAV-AB and STAV-FCS cell lines.

### Transmission electron microscopy

Xenografts were dissected and small pieces were fixed in 2% glutaraldehyde + 0.5% paraformaldehyde in 0.1 M sodium cacodylate buffer containing 0.1 M sucrose and 3 mM CaCl_2_, pH 7.4, at room temperature for 30 min followed by 24 h at 4°C. Specimens were then rinsed in 0.1 M phosphate buffer, pH 7.4, postfixed in 2% osmium tetroxide 0.1 M phosphate buffer, pH 7.4, at 4°C for 2 h, dehydrated in ethanol followed by acetone, and embedded in LX-112 (Ladd, Burlington, VT, USA). Semithin sections were cut and stained with toluidine blue and used for light microscopic analysis. Ultrathin sections (∼40–50 nm) were cut and contrasted with uranyl acetate followed by lead citrate and examined in a Leo 906 transmission electron microscope at 80 kV (Leo, Oberkochen, Germany).

### Ethical approval

This study was approved by the regional Animal Experiment Ethics Review Committee of Northern Stockholm (N19/05).

## Results

### Growth characteristics of xenografts

Following inoculation of STAV-AB and STAV-FCS cells into SCID mice, STAV-FCS xenografts had a considerably higher growth rate than those derived from STAV-AB cells. Tumor formation of the STAV-FCS cells could be detected by palpation after 12 weeks. For STAV-AB, 14 weeks were required before subcutaneous tumors could be detected. Experiments with shorter incubation periods showed no tumor formation.

Tumor take after 15 weeks was 3/10; two of five mice inoculated with STAV-FCS and one of five with STAV-AB cells. In the second generation, tumor take was 3/3 for STAV-FCS and 2/3 for STAV-AB (Figures 1B,C). At serial passage, STAV-FCS cells gave rise to rapidly growing tumors that were detected already after 1 week, whereas STAV-AB xenografts were palpable only after 4 and 4.5 weeks after inoculation and grew slower (Figures 1B,C). Xenografts from STAV-FCS cells grew faster than STAV-AB, enabling a third passage to be performed with the STAV-FCS cells.

### Xenografts of both cell sub-lines converged to a similar epithelioid-like morphology

**Figure 2:**
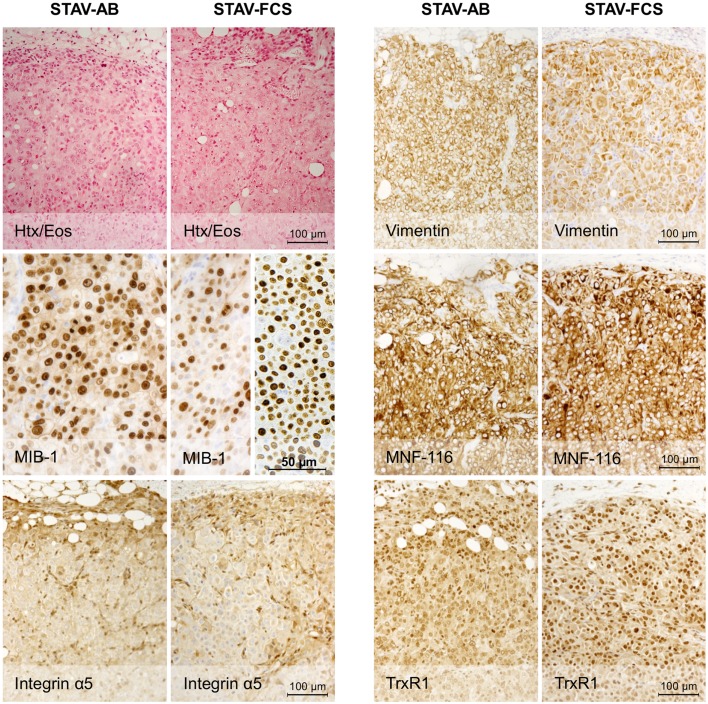
**Histological analysis of xenografts and immunohistochemical staining for MIB-1, Integrin α5, and epithelial and mesenchymal markers**. Hematoxylin/eosin staining revealed a similar epithelioid phenotype in xenografts originating from both STAV-AB and STAV-FCS cell sub-lines. MIB-1 staining indicated proliferative activity in both xenografts, however, less vascularized areas of STAV-FCS xenografts (left sub-panel) showed lower labeling index than more vascularized peripheral areas (right sub-panel). Expression of vimentin, a mesenchymal differentiation marker, was strong in xenografts from both cell sub-lines, as was expression of the integrin α5 subunit. Similarly, MNF-116, an epithelial differentiation marker, was strongly expressed in xenografts from both cell sub-lines. TrxR1 showed a difference in staining pattern between xenografts of the two cell sub-lines, where STAV-FCS-derived xenografts showed a stronger nuclear immunoreactivity. STAV-AB-derived xenografts also, however, stained positively for TrxR1.

**Figure 3:**
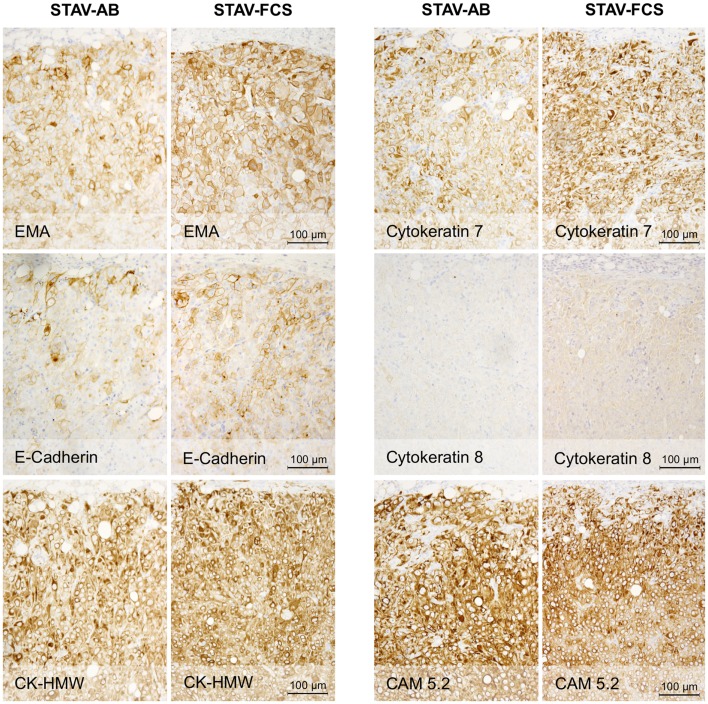
**Immunohistochemical staining of xenografts for markers of epithelial differentiation**. Xenografts from both cell sub-lines stained uniformly and similarly positive for EMA, E-cadherin, CK-HMW, CK7, and CAM 5.2, and negative for CK8. These proteins are markers for epithelioid differentiation.

**Figure 4:**
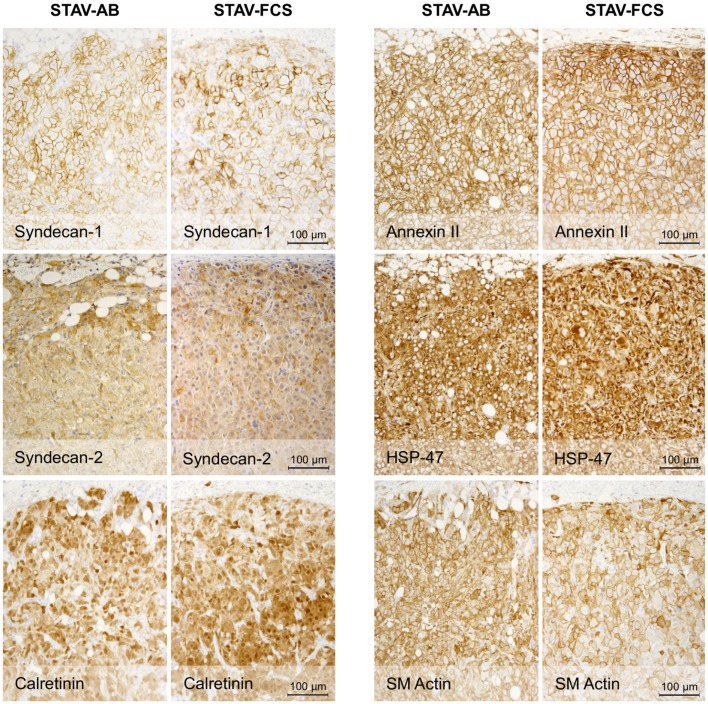
**Immunohistochemical staining of xenografts for mesothelial and mesenchymal differentiation markers**. Xenografts from both cell sub-lines stained uniformly and similarly positive for Syndecan-1, Syndecan-2, Calretinin, Annexin-II, HSP47, and SM actin.

Light microscopy of xenografts showed a similar, epithelioid-like morphology regardless of the original phenotype of the inoculated cell sub-lines (Figure 2). Remarkably, xenografts derived from both STAV-AB and STAV-FCS cells showed similar staining patterns for most of the tested antigens, comprising a broad range of mesothelial, epithelial, and mesenchymal markers (Figures 2–4; Table 1). Integrin αVβ5 stained positively in xenografts from both cell sub-lines, with a modest accentuation at the tumor invasion front of the STAV-AB-derived xenografts (Figure 2).

**Table 1:** **Immunohistochemical analyses of xenografts**.

	STAV-AB xenograft	STAV-FCS xenograft	Localization
**MESOTHELIAL AND EPITHELIAL MARKERS**			
Calretinin	++	++	Cytoplasm and nucleus
EMA	+	+(+)	Cell membrane
E-cadherin	+ (Focal)	+	Cell membrane
CK-HMW	++	++	Cytoplasm
Cytokeratin 7	+	++	Cytoplasm
Cytokeratin 8	−	−	Cytoplasm
Cam 5.2	+++	++	Cell membrane and cytoplasm
Syndecan-1	+	+	Cell membrane
MNF-116	+++	+++	Cell membrane and cytoplasm
**DIFFERENTIATION-RELATED MARKERS**			
TrxR1	++	+++	Cytoplasm and nucleus
Integrin α5	+	+	Cytoplasm
HSP-47	+++	+++	Cell membrane and cytoplasm
Annexin-II	++	++	Cell membrane
**MESENCHYMAL MARKERS**			
Vimentin	++	++	Cytoplasm
SM actin	+(+)	+	Cytoplasm
Syndecan-2	++	++	Cytoplasm
**PROLIFERATION MARKER**			
Mib-1	>80%	40%	Nucleus

**Figure 5:**
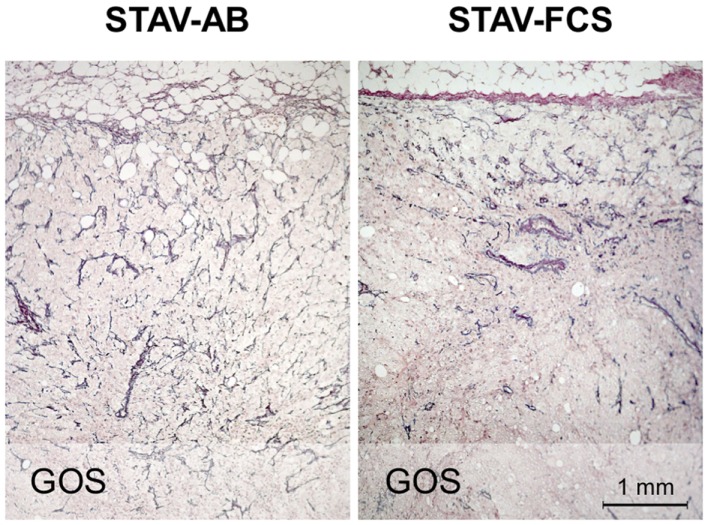
**Microvessel density in xenografts demonstrated by staining for reticulin fibers (Gordon and Sweets)**. Xenografts from STAV-AB cells showed uniform vascularization, whereas xenografts from STAV-FCS cells showed decreased vascularization at a depth of 0.5–1 mm into the tumor. The tumor invasion front is seen at the top in both images.

MIB-1 staining indicated that both xenografts were highly proliferative. Despite that STAV-FCS-cell-derived xenografts grew faster and reached the maximal size earlier in SCID mice, this tissue showed areas with reduced MIB-1 labeling index. These areas of less proliferation were mainly seen in the central parts of xenografts, corresponding to decreased vascularization, and areas of necrosis. Both microvessel density and vessel volume density, as demonstrated in the reticulin stained samples, were significantly greater close to the infiltration front, while only a few vessels were seen deeper than 1.2 mm in the tumor tissue (Figure 5; Tables 3 and 4). At distance from the tumor front the sarcomatoid cell xenografts contained significantly less vessels compared to the corresponding areas in the epithelioid cell xenografts, and the difference between surface and deeper layers was most apparent in the sarcomatoid cell STAV-FCS preparation.

TrxR1, which in culture is more expressed in the STAV-AB cell line, showed greater reactivity in the STAV-FCS-derived xenografts, and this reactivity was mainly nuclear (Figure 2; Table 1). No immunoreactivity was seen when using goat serum and isotype IgG controls (data not shown).

### FISH analyses demonstrated heterogeneous cell populations

Cytogenetic analysis by FISH proved that both the original cell lines and the derived xenografts were heterogeneous. FISH analysis by the UroVysion probe set and four additional PAC/BAC probes located on chromosome 3 showed maintained heterogeneous subpopulations at around 10% for STAV-AB and around 20% for the FCS cell line. In the xenografts, the UroVysion probe set indicated that 20% of the cells in STAV-AB derived xenografts and around 30% of the cells in STAV-FCS derived ones, represented non-dominant clones.

### Electron microscopy demonstrated epithelioid phenotypic traits in xenografts

**Figure 6:**
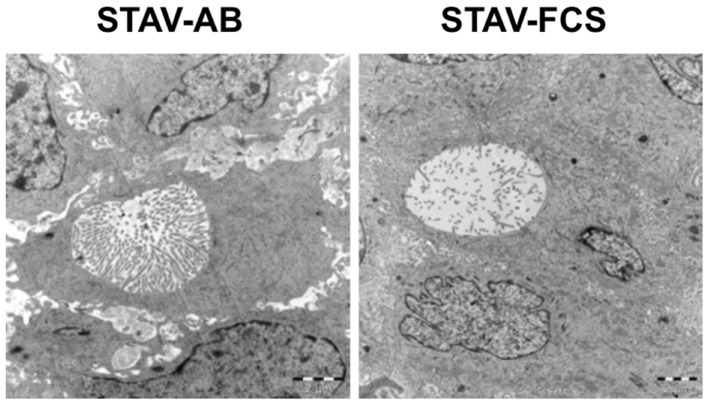
**Ultrastructural characterization of xenografts**. Electron microscopy revealed the presence of microvilli-laden neolumina in xenografts from both phenotypes. A greater density of microvilli was consistently found in the STAV-AB-derived xenografts. The density of microvilli was strikingly lower in the STAV-FCS-derived xenografts, suggesting that the cells displayed a less differentiated state. Scale bars are 2 μm.

In earlier *in vitro* studies, STAV-AB and STAV-FCS cells showed robust phenotypic differences consistent with their respective differentiation patterns (10). Characteristic ultrastructural features of both xenografts were that of an epithelioid subtype of malignant mesothelioma, as evidenced by desmosomes, numerous slender, long microvilli on cell surfaces and in intracytoplasmic neolumina, and absence of glycocalyx. Interestingly, the density of microvilli was lower in the STAV-FCS-derived xenografts (Figure 6), indicating that the cells displayed a less differentiated morphology.

### Xenografts of both cell sub-lines converged toward a similar genotype resembling the original sarcomatoid cells

**Figure 7:**
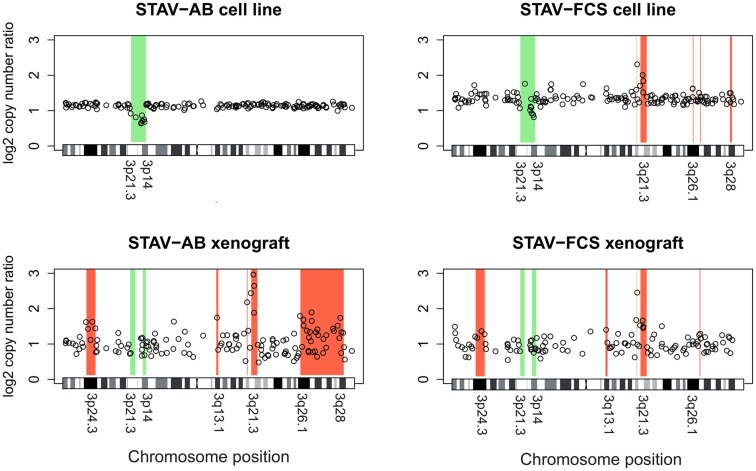
**Array comparative genomic hybridization analysis showing gains and losses on chromosome 3 in cell lines and xenografts**. Regions indicative of gain are highlighted in red, whereas regions indicative of loss are highlighted in green. Xenografts from both cell sub-lines showed a similar pattern of gains and losses, except for a large terminal region on the q arm gained in STAV-AB-derived xenografts. Compared to the cell sub-lines, xenografts irrespective of origin were more similar to STAV-FCS cells, suggesting a clonal expansion in the STAV-AB-derived xenografts of cells with genetic similarities to STAV-FCS cells. The variability was greater in xenografts, likely because those specimens were fixed in formaldehyde.

Chromosome 3 array CGH analysis was performed on the original STAV-AB and STAV-FCS cell sub-lines and the derived xenografts. Irrespective of their original cell sub-line, xenografts derived from both cell sub-lines showed a similar pattern of chromosome 3 aberrations rather resembling the sarcomatoid STAV-FCS cells (Figure 7; Table 2).

**Table 2:** **Regions of chromosome 3 and gene locations**.

Chromosome band	FISH clone	Genes	Gain/loss			
			STAV-AB cell line	STAV-FCS cell line	STAV-AB xenograft	STAV-FCS xenograft
3p24.3	RP11-255o19–RP11-208g16	KAT2B, VENTXP7, RAB5A, KCNH8, SATB1, PLCL2, RFTN1, GALNTL2, DPH3			Gain	Gain
3p21.3–3p14	RP11-91p19–RP11-169g24	LTF, TDGF1, WNT5a, RASSF1, CACNA2D3, GNAI2, SEMA3B	Loss	Loss	Loss	Loss
3q13.1	RP11-90i19	No genes			Gain	Gain
3q21.2–3q22.1	RP11-711f17, PR11-124n2; RP11-177o2–RP11-893g19	ITGB5, SNX4, OSBPL11, ROPN1B, ALD1HL1, KLF15, RAB7, MGLL, RUVBL1, H1X, H1FOO, MBD4, IFT122, TRH		Gain	Gain	Gain
3q26.2–3q28	RP11-24l16–RP11-79o17	EVI1, TP63, LPP		Gain	Gain	Gain

**Table 3:** **Volume density of blood vessels in xenografts**.

Distance from tumor margin (mm)	Mean density ± SE	
	STAV-AB	STAV-FCS
0–1.2	0.083 ± 0.019	0.096 ± 0.020
1.2–4	0.028 ± 0.007	0.020 ± 0.006

**Table 4:** **Microvessel density in xenografts (vessels/mm^2^)**.

Distance from tumor margin (mm)	Mean density ± SE	
	
	STAV-AB	STAV-FCS
0–1.2	14.3 ± 1.7	16.0 ± 1.8
1.2–4	6.8 ± 0.8	3.2 ± 0.5

The original STAV-AB and STAV-FCS cell sub-lines shared a common deletion at 3p14.3p21.3 (Figure 7; Table 2). Within this region only six clones were lost in the xenografts. Copy number gains were seen in the sarcomatoid STAV-FCS cell sub-line and in both xenografts. Three such shared regions with increased copy number were seen predominantly on the 3q arm, comprising the 3q21.2–3q22.1, and 3q26.2 regions. One clone (PR11-124n2) showed copy number gain both in the STAV-FCS cell sub-line and its xenograft, but was not present in the STAV-AB cells and its derived xenograft, which makes it an FCS specific clone and a possible candidate that might have role in epithelial-mesenchymal transition. Interestingly, the 3p24.3 and 3q13.1 regions were found to harbor copy number gains exclusively in the xenografts, whereas they were not detected in the original cell sub-lines. The STAV-AB xenograft showed elevated binding intensities in an area spanning 30 MB at 3q26.1–3q28. Some of the clones from this region (RP11-24l16–RP11-79o17) were found in copy number gain also in the STAV-FCS cell sub-line and its xenograft.

## Discussion

Mesothelioma cells are able to differentiate along the mesenchymal to epithelial axis, and in biphasic tumors epithelioid and sarcomatoid cells are present side by side in the same tumor. Mesothelioma differentiation is a critical factor influencing clinical outcome and treatment response (11–13). Prognosis deteriorates dramatically when the sarcomatous phenotype is dominant. However, little is known about the factors regulating this differentiation between phenotypes. The genetic background and microenvironmental clues underlying this process are also poorly understood, and relevant animal models addressing phenotypic changes and molecular switches associated with this process are sparse. In this study we describe the establishment of a xenograft model to study malignant mesothelioma and we propose genes potentially involved in epithelial or mesenchymal differentiation.

In cell culture, the two cell sub-lines used in this study have stable phenotypes with epithelioid and sarcomatoid growth patterns, distinct immunophenotypes, and molecular signatures (14), recapitulating *in vitro* the major morphological features of malignant mesothelioma. In the present study, we demonstrate tumor cell heterogeneity within each original cell sub-line, with the largest non-dominant subpopulation found in the STAV-FCS cells. Chromosome 3 CGH arrays indicated different dominating patterns for the two sub-lines with a common deleted region at 3p14–21.3. This deletion was the only change in the epithelioid cells, while the sarcomatoid cells showed five additional amplified regions on 3q (Figure 7; Table 2).

Following inoculation in SCID mice, both phenotypes were able to initiate tumor growth. However, the tumor take was initially low, and similarly to the clinical course of mesothelioma a long latency period was required before tumor formation could be seen. Serial passages resulted in a high take rate and faster tumor formation. The low initial take rate is comparable to that seen in other studies of mesothelioma xenografts (36), and suggests clonal expansion from a precursor subpopulation, originally being present as minor proportions in the inoculated cell sub-lines, as supported by findings of heterogeneity using FISH. The original heterogeneity seen by cytogenetics was maintained in the xenografts, as evidenced by an increased proportion of heterogeneous subpopulation in the STAV-FCS-derived xenografts compared to the STAV-AB-derived ones.

Xenografts derived from the two cell sub-lines converged to a similar morphology with predominantly epithelioid characteristics, as evidenced by both light and electron microscopy. Immunohistochemical analysis of differentiation markers confirmed this similarity between the two xenografts, with broadly similar expression levels of all investigated differentiation markers. Markers for both epithelial and mesenchymal differentiation were found to be positive, suggesting an intermediate phenotype although the morphological characteristics resembled polygonal epithelioid differentiation.

The convergent phenotypes observed in xenografts may be explained by clonal expansion under a selection pressure in the SCID mouse microenvironment, favoring cells with the most prevalent sarcomatoid STAV-FCS chromosome 3 genotype, and harboring common amplified regions on 3q. Although the morphologic phenotype of the expanding cell clones were quite similar, their respective growth rates differed considerably. Cells from the sarcomatoid cell sub-line formed a 1 cm^3^ nodule much faster than the epithelioid cells, also in the second generation of xenograft. However, the total proliferation rate, measured in the harvested xenograft as proportion of MIB-1 reactive cells, was lower in the faster growing xenograft derived from sarcomatoid cells. To further investigate this contradictory result, we analyzed microvessel density. While the sarcomatoid cell-derived xenograft had a slightly higher density at the infiltration front compared to the corresponding epithelioid cell xenograft, the corresponding density in the deeper parts in the sarcomatoid cell-derived xenograft was much lower with an obvious tendency to necrosis in the tissue. Thus, an insufficient vascularization of the sarcomatoid xenograft, in relation to its growth rate, is a possible explanation for the discrepancy between macroscopic growth and MIB-1 expression.

Cytogenetic analysis of chromosome 3 showed that xenografts derived from both cell lines had similar genotypes, most closely resembling the more aggressive sarcomatoid cell sub-line. FISH of chromosome 3 demonstrated genetically heterogeneous cell populations in all cell lines and xenografts. We propose that this heterogeneity may result in plasticity in differentiation patterns. Different microenvironmental pressures may then cause preferential expansion of certain subpopulations. Certain genotypic changes, manifested in this study as chromosome 3 rearrangements, might be characteristics of the mesenchymal phenotype (e.g., the clone RP11-124n2 at 3q21) or others of a more aggressive sarcomatoid component of malignant mesotheliomas (clones amplified in STAV-FCS and both xenografts). We can, however, not rule out contributions to this process from epigenetic changes induced by the microenvironment nor from *de novo* mutations.

The four different aCGH patterns have one region suggestive of a deletion on 3p in common. This may represent an early aberration necessary for the malignant phenotype and for the ability of cells to survive in culture. The region located between 3p21.3 and 3p14.3, deleted in both cell sub-lines, contains several established tumor suppressor genes including LTF, TDGF1, WNT5a, and RASSF (37), as well as candidate tumor suppressors such as CACNA2D3, GNAI2, and SEMA3B. The telomeric breakpoint of this region coincides with a previously described tumor breakpoint region with instability characteristics (38). Breaks at this region, containing special DNA structures with large segmental duplications conferring chromosomes special features of instability, observed in certain tumors, suggest a more aggressive genotype. Rearrangement at this region was shown to be characteristic for tumors with high plasticity/rearrangement rate and more aggressive genotype (29). These findings fit the known natural history of sarcomatoid vs epitheloid phenotypes of malignant mesothelioma. Some clones within this region were also lost in the xenografts as a hallmark of their clonal outgrowth, presumably due to selective pressure in the SCID mouse microenvironment. These two deleted regions at 3p21.3 in all cell lines and xenografts are consistent with characteristic genetic changes in malignant mesothelioma (39–41).

There were three common regions suggestive of increased copy number in sarcomatoid cells and both xenografts, in which some genes are of particular interest. The 3q21.3 region contains several putative and reported oncogenes/tumor promoting genes. Genes present in this region have functions in DNA-binding and transcription.

A region at 3q26.1 suggestive of common amplification harbors genes with functions in DNA-binding, transcription, and oncogenic transformation (see Table 2). This region contains the EVI1 oncogene, which among other functions can destabilize chromosomal organization (42). Another region at 3q26.1 does not contain any gene but has small miRNAs. Regions between RP11-178k17 and RP11-54l9 contain the TP63 gene at 190 Mb, which is a tumor promoting gene even though it belongs to the p53 family. P63 acts primarily through negative regulation by theDeltaNp63/p73 isoforms to regulate cell death and differentiation (43).

The long incubation times needed to obtain tumor takes suggest that the acquired xenografts represent clonal outgrowths from cells representing a fraction of the original cell sub-lines. The phenotypic similarity of cells originally derived from dissimilar differentiation states demonstrates the importance of evolutionary selection pressures in the mouse microenvironment. The chromosomal rearrangements studied were also more similar between xenografts than between the two original cell cultures. The cells from which the xenografts originates may therefore represent a stem cell-like subpopulation of tumor cells, more closely related to each other than to the dominating cell subpopulations.

Major phenotypic differences were not seen between xenografts, but the sarcomatoid mesothelioma cells had a faster growth *in vivo* which mirrors the more aggressive biological behavior of this tumor. The different growth patterns in mixed type mesotheliomas could be a result of clonal outgrowth, which in turn depends on the microenvironment in the tumor. The present *in vitro* and corresponding *in vivo* model of malignant mesothelioma is well characterized and can be used for studying the interplay of environmental factors and the clonal evolution of a heterogeneous tumor population.

## Conflict of Interest Statement

The authors declare that the research was conducted in the absence of any commercial or financial relationships that could be construed as a potential conflict of interest.

## Footnotes

^1^http://bacpac.chori.org/

^2^http://genome.ucsc.edu/

^3^http://www.ncbi.nlm.nih.gov/geo/query/acc.cgi?acc=GSE48019
